# Synthesis of Novel *E-*2-Chlorovinyltellurium Compounds Based on the Stereospecific *Anti*-addition of Tellurium Tetrachloride to Acetylene

**DOI:** 10.3390/molecules17055770

**Published:** 2012-05-15

**Authors:** Maria V. Musalova, Vladimir A. Potapov, Svetlana V. Amosova

**Affiliations:** A. E. Favorsky Irkutsk Institute of Chemistry, Siberian Branch of the Russian Academy of Sciences, 1 Favorsky Str., Irkutsk 664033, Russia

**Keywords:** acetylene, tellurium tetrachloride, *anti*-addition, chlorovinyl tellurides, chlorovinyltellurium trichlorides, bis(2-chlorovinyl)tellurium dichlorides, divinyl ditellurides

## Abstract

The reaction of tellurium tetrachloride with acetylene proceeds in a stereospecific *anti*-addition manner to afford the novel products *E*-2-chlorovinyltellurium trichloride and *E*,*E*-bis(2-chlorovinyl)tellurium dichloride. Reaction conditions for the selective preparation of each of these products were found. The latter was obtained in 90% yield in CHCl_3_ under a pressure of acetylene of 10–15 atm, whereas the former product was formed in up to 72% yield in CCl_4_ under a pressure of acetylene of 1–3 atm. Synthesis of the previously unknown *E,E*-bis(2-chlorovinyl) telluride, *E*,*E*-bis(2-chlorovinyl) ditelluride, *E*-2-chlorovinyl 1,2,2-trichloroethyl telluride and *E*,*E*-bis(2-chlorovinyl)-tellurium dibromide is described.

## 1. Introduction

Selenium was considered a poison for many years, until Schwarz and Foltz identified it as an essential micronutrient for mammals, including human beings [1]. Like selenium, tellurium was regarded a poison for many years until non-toxic organotellurium compounds with high biological activity were found [2,3,4,5,6,7,8]. Like organoselenium compounds, a number of organotellurium compounds exhibit high glutathione peroxidase-like activity [2,3,4,5]. A four-valent tellurium compound, ammonium trichloro(dioxoethylene-O,O′-)tellurate, also known as AS-101, possesses high immunomodulating activity [2,3,6,7,8]. When administered to mice, this compound mediates antitumor effects and protects mice from ionizing radiation [2,3,6]. The tests demonstrated that AS-101 is potentially useful in the treatment of clinical immunosuppression conditions involving cancer and AIDS [2,3,6]. The literature [2,3,4,5,6,7,8] indicates that among organotellurium compounds, mainly telluranes (four-valent tellurium compounds), exhibit high biological activity. 

The principal electrophilic tellurium-containing reagent is tellurium tetrachloride. The first example of the addition of TeCl_4_ to acetylenes was reported in 1962 [9]. The reaction of TeCl_4_ with phenylacetylene and diphenylacetylene afforded the corresponding 2-chlorovinyltellurium trichlorides, however, the stereochemistry of the products was not determined [9]. It has been shown later that the reactions of TeCl_4_ with phenylacetylene, diphenylacetylene and alkylphenylacetylenes proceed in highly regiospecific and stereospecific manner via *syn*-addition to afford the products of *Z*-stereochemistry [10,11,12,13,14,15]. The *Z*-configuration was confirmed by X-ray analysis [15]. A special case is the addition of tellurium tetrachloride to acetylenic alcohols [13,16], since the hydroxy group influences the stereochemistry [13].

Nowadays, the distinguishing property of tellurium reagents to react with high regio- and stereoselectivity finds increasing application in organic synthesis [17,18,19]. The adducts of TeCl_4_ with acetylenes were recognized as important precursors and synthons for organic synthesis and applied in many approaches for the preparation of various functionalized alkenes in a highly regio- and stereospecific manner [17,18,19]. The reduction of bisadducts of TeCl_4_ with acetylenes gives 2-chloro-vinyltellurides, which are used for preparation of various useful products by metallation and cross-coupling reactions [11,17,18,19].

**Scheme 1:**
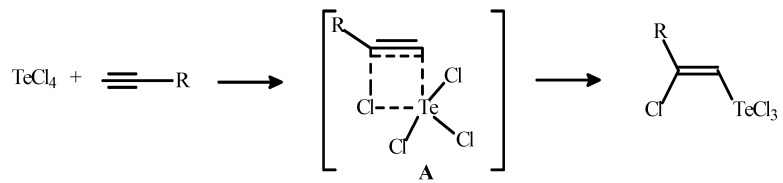
The mechanism of stereospecific *syn*-addition of TeCl_4_ to alkynes.

A mechanism with the formation of 4-membered transition state **A** was proposed in order to explain stereospecific *syn*-addition of tellurium tetrachloride to the triple bond of alkynes (Scheme 1) [14,20].

It is worth noting that compounds with high biological (antioxidative and antimetastatic) activity were found among the adducts of TeCl_4_ with acetylenes [3]. Therefore, studies of previously unknown reactions of tellurium tetrachloride with acetylenes with the goal to obtain novel organotellurium (IV) compounds and to investigate their properties is an important task for organic chemists.

Synthesis of unsaturated tellurides and studies of their properties is the subject of our continued interest [21,22,23,24,25,26,27,28,29,30]. Earlier we elaborated efficient methods for the preparation of vinylic tellurides by nucleophilic addition of telluride and organotellurolate anions to acetylene [31,32,33,34,35] and phenylacetylene [36,37,38]. Addition of tellurium tetrachloride to trimethylethynyl silane and diorganyl-diethynyl silanes led to novel unsaturated tellurium-silicon containing compounds [39,40]. The present paper describes electrophilic addition of tellurium tetrachloride to acetylene. 

Acetylene is a versatile multi-thousand ton chemical feedstock and many its reactions are of high value from both theoretical and practical viewpoints. In view of the rapid depletion of hydrocarbon resources, acetylene, which can be alternatively manufactured from coal, is expected to acquire an increasingly more important role as a universal chemical starting material [41]. Reactions of inorganic compounds with acetylene by convenient procedures giving high yields of target products may find useful applications, not only in organic synthesis, but in industry as well. 

## 2. Results and Discussion

There were no data in the literature concerning reactions of tellurium halides with unsubstituted acetylene prior to our research. In a letter [42] we briefly reported our preliminary results on studies of the reaction of tellurium tetrachloride with acetylene and the formation of a bisadduct, the previously unknown *E*,*E*-bis(2-chlorovinyl)tellurium dichloride (**1**), in 62% yield. The present paper is the complete account of our studies of this reaction under various reaction conditions. 

**Scheme 2:**
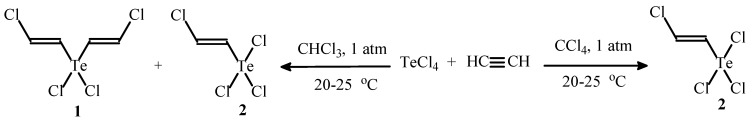
Reactions of TeCl_4_ with acetylene under atmospheric pressure.

We have found that the performance of the reaction of tellurium tetrachloride with acetylene in carbon tetrachloride at room temperature under atmospheric pressure allows one to obtain a monoadduct, the previously unknown *E*-(2-chlorovinyl)tellurium trichloride (**2**) (Scheme 2). 

In carbon tetrachloride, the reaction proceeded chemo- and stereoselectively via *anti*-addition to give only monoadduct **2** and the formation of bisadduct **1** was not observed. When acetylene was bubbled into the reaction mixture at room temperature during 2 h, the yield of monoadduct **2** was 30% (with incomplete conversion of tellurium tetrachloride). Increasing the duration of the reaction from 2 to 5 h raised the conversion of tellurium tetrachloride, but led to the formation of some by-products. 

Monoadduct **2** is also formed when the reaction was carried out in chloroform at room temperature under atmospheric pressure, but compound **2** was converted into bisadduct **1** (82% yield) under these conditions (Scheme 2). The selective formation of compound **2** was observed in the initial period of the reaction in chloroform (^1^H-NMR monitoring of the reaction carried out in CDCl_3_); then bisadduct **1** appeared and its content increased over time and, consequently, the concentration of monoadduct **2** decreased in time and, finally, disappeared by the end of the reaction.

Performing the reaction of tellurium tetrachloride with acetylene in carbon tetrachloride at a temperature of 10–20 °C under a pressure of acetylene of 2–3 atm in an autoclave allowed us to increase the conversion of TeCl_4_ and to obtain compound **2** in 72% yield. 

**Scheme 3:**
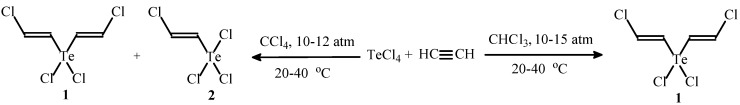
Reactions of TeCl_4_ with acetylene under the pressure in an autoclave.

The highest yield of bisadduct **1** was achieved when the reaction of tellurium tetrachloride with acetylene was carried out in an autoclave under a higher pressure of acetylene (12–15 atm) in dry chloroform at 20–40 °C for 5 h (Scheme 3). The reaction proceeds in a stereospecific manner via *anti-*addition to afford the product **1** in 94% yield. 

When the reaction of tellurium tetrachloride with acetylene was carried out in carbon tetrachloride under a pressure of acetylene of 10–12 atm at room temperature in an autoclave, the formation of both products **1** and **2** in 78% and 17% yields, respectively, was observed (Scheme 3). Increasing the pressure of acetylene to 14–15 atm and the reaction temperature to 30–40 °C permitted us to selectively obtain bisadduct **1** in 90% yield.

**Scheme 4:**
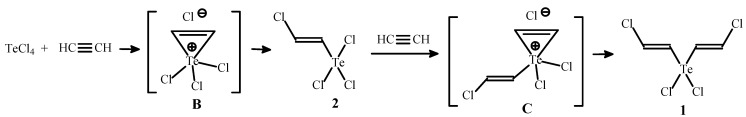
The supposed mechanism of the *anti-*addition of TeCl_4_ to acetylene.

Thus, in contrast to the *syn-*addition of tellurium tetrachloride to substituted acetylenes [9,10,11,12,13,14,15], the reaction of tellurium tetrachloride with acetylene proceeds as an *anti*-addition to give the products **1** and **2** of *E*-stereochemistry. This is the first example of the *anti*-addition of tellurium tetrachloride to acetylenic hydrocarbons. The formation of the 3-membered intermediates **B** and **C** was supposed to explain the stereospecific *anti*-addition (Scheme 4). 

We presume that in the case of acetylene, the 3-membered intermediates (**B** and **C**, Scheme 4) are energetically preferred in comparison with a possible 4-membered transition state **A** (Scheme 1). It is noteworthy that the formation of similar 3-membered intermediates is well known for the addition of organic sulfenyl and selenenyl halides (RSHal, RSeHal) to alkynes [43]. 

**Scheme 5:**
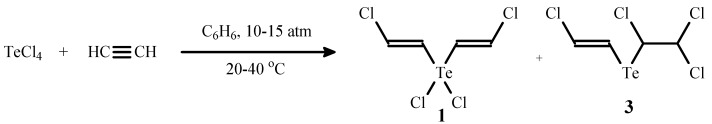
The reaction of TeCl_4_ with acetylene in benzene.

When the reaction was carried out in benzene under similar conditions (25–40 °C, autoclave, pressure of acetylene of 10–15 atm), the formation of *E*-2-chlorovinyl 1,2,2-trichloroethyl telluride (**3**) in 10–15% yield was observed, along with the main product **1** (70–82% yield) (Scheme 5).

It would be logical to suppose that the formation of **3** is the result of rearrangement of chlorine atoms in compound **1**. However, heating bisadduct **1** in benzene at 40 °C did not give compound **3**. 

**Scheme 6:**
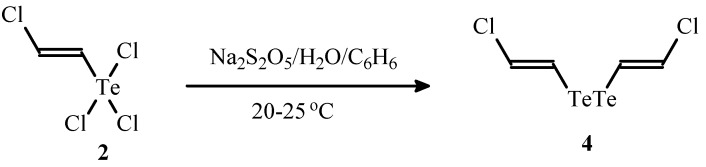
The synthesis of ditelluride **4** by reduction of compound **2**.

Previously unknown *E*,*E*-bis(2-chlorovinyl) ditelluride (**4**) was obtained in 64% yield by the reduction of compound **2** with aqueous solution of Na_2_S_2_O_5_ (Scheme 6). 

Organic ditellurides constitute an important class of organotellurium compounds, which are used for generation of highly nucleophilic organyltellurolate anions by reduction of the Te-Te bond as well as for preparation of electrophilic reagents RTeHal and RTeHal_3_ by halogenations (SO_2_Cl_2_, Br_2_) of organic ditellurides [17,18,19]. Compound **4** can find application as a starting material for the synthesis of novel compounds bearing an *E*-2-chlorovinyltellanyl moiety. It is noteworthy that data on synthesis of divinyl ditellurides are very scarce in the literature [44,45,46]. 

**Scheme 7:**
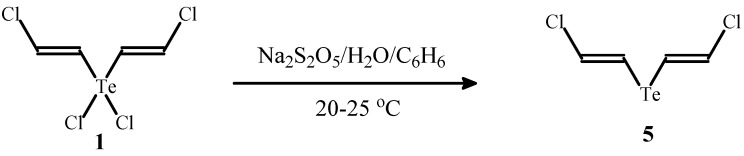
The synthesis of telluride **5** by reduction of compound **1**.

In a similar manner, bisadduct **1** was reduced to previously unknown *E*,*E*-bis(2-chlorovinyl) telluride (**5**) in 86% yield (Scheme 7).

**Scheme 8:**
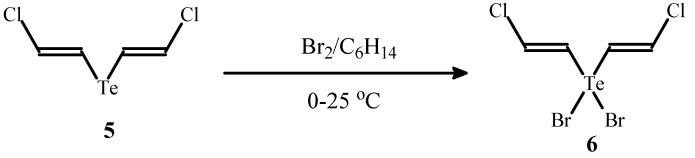
The synthesis of tellurane **6** by the reaction of telluride **5** with bromine.

It is known that 2-halovinyltellurides are used for the preparation of valuable products by cross-coupling reactions [17,18,19] and therefore telluride **5** may be useful for stereoselective synthesis of functionalized alkenes. The reaction of telluride **5** with bromine afforded tellurane **6** containing different halogen atoms in one molecule (Scheme 8).

## 3. Experimental

### 3.1. General Information

^1^H (400.1 MHz) and ^13^C (100.6 MHz) spectra were recorded on a Bruker DPX-400 spectrometer (Karlsruhe, Germany) in DMSO-*d_6_*, or CDCl_3_ (HMDS). Mass spectra were recorded on a Shimadzu GCMS-QP5050A spectrometer (Duisburg, Germany). Tellurium tetrachloride was prepared from tellurium and sulfuryl chloride [12].

### 3.2. Synthetic Procedures for the Preparation of Compounds *1–6*

*E*,*E-Bis(2-chlorovinyl)tellurium dichloride* (**1**). A mixture of TeCl_4_ (2.69 g, 10 mmol) and dry chloroform (100 mL) was heated (30–40 °C) in a 1 L rotating autoclave under acetylene pressure (12-14 atm) for 5 h. The solvent was evaporated and the residue was washed with cold hexane and dried to give compound **1** (3.03 g, 94% yield) as a colorless powder, m.p. 112–113 °C. Found (%): C, 15.24; H, 1.17; Cl, 43.89. C_4_H_4_Cl_4_Te. Calculated (%): C, 14.94; H, 1.25; Cl, 44.11. ^1^H-NMR (DMSO-*d_6_*, δ, ppm): 7.25 (d, 2H, *J* = 13.9 Hz), 7.48 (d, 2H, *J* = 13.9 Hz). ^13^C-NMR (DMSO-*d_6_*, δ, ppm): 129.80 (TeCH), 133.34 (CHCl). MS (70 eV), *m/z* (*I*_rel._, %): 287 (100) [M-Cl]^+^, 252 (48) [M-2Cl]^+^, 226 (71) [ClTeCH=CHCl]^+^, 200 (12) [TeCl2]^+^, 191 (28) [TeCH=CHCl]^+^, 165 (66) [TeCl]^+^, 130 (52) [Te]^+^, 122 (82) [C_4_H_4_Cl_2_]^+^, 87 (86) [C_4_H_4_Cl]^+^, 61 (37) [C_2_H_2_Cl]^+^, 51 (70) [C_4_H_3_]^+^, 36 (52) [HCl]^+^, 26 (80) [C_2_H_2_]^+^. 

*E-(2-Chlorovinyl)tellurium trichloride *(**2**). Dry CCl_4_ (50 mL) was saturated with acetylene by bubbling dry acetylene for 1 h at atmospheric pressure. Then TeCl_4_ (0.54 g, 2 mmol) was added and dry acetylene was bubbled through the mixture with intensive stirring for 2 h. The solution was decanted from the precipitate and new portion of dry CCl_4_ (50 mL) was added to the precipitate and stirred for 1 h. The solution was decanted from the precipitate and combined with the first portion of the CCl_4_ solution. After evaporation of the solvent, the residue was washed with cold hexane and dried to give product **2** (0.18 g, 30% yield)—Colorless powder, which upon heating begins to darken at 45–47 °C and then decomposed. Found (%): C, 7.98; H, 0.78; Cl, 47.66. C_2_H_2_Cl_4_Te. Calculated (%): C, 8.13; H, 0.68; Cl, 48.00. ^1^H-NMR (DMSO-*d_6_*, δ, ppm): 7.38 (d, 2H, CHCl, *J* = 13.4 Hz), 7.82 (d, 2H, TeCH,*J* = 13.4 Hz). ^13^C-NMR (DMSO-*d_6_*, δ, ppm): 131.32, 145.67. 

*E-2-Chlorovinyl 2*,*2*,*1-trichloroethyl telluride *(**3**). A mixture of TeCl_4_ (2.69 g, 10 mmol) and benzene (80 mL) was heated (20–40 °C) in a 1 L rotating autoclave under acetylene pressure (12–14 atm) for 5 h. The solvent was evaporated and the residue was washed with cold hexane and dried to give compound **1** (2.25 g, 70% yield) as a colorless powder. The solvent was evaporated from the hexane solution and the residue was subjected to short column chromatography on silica gel (eluent hexane) to give compound **3** (0.48 g, 15% yield) as a dark liquid. Found (%): C, 14.58; H, 1.36; Cl, 44.45. C_4_H_4_Cl_4_Te. Calculated (%): C, 14.94; H, 1.25; Cl, 44.11. ^1^H-NMR (CDCl_3_, δ, ppm): 5.32 (d, 1H, *J* = 3.3 Hz); 6.03 (d, 1H, *J* = 3.3 Hz), 6.66 (d, 1H, *J* = 13.3 Hz), 6.87 (d, 1H, *J* = 13.3 Hz). ^13^C-NMR (CDCl_3_, δ, ppm): 62.89 (TeCHCl), 74.58 (CHCl_2_). 116.21 (TeCH=), 127.87 (=CHCl).

*E*,*E-Bis(2-chlorovinyl) ditelluride *(**4**). A solution of Na_2_S_2_O_5_ (0.95 g, 5 mmol) in water (5 mL) was added to a mixture of compound **2** (0.15 g, 0.5 mmol) and benzene (2 mL). The resulted mixture was vigorously stirred at room temperature for 24 h under argon. The mixture was extracted with benzene (3 × 5 mL), organic phase was dried, filtered and the solvent was evaporated. The residue was subjected to column chromatography (eluent–hexane) to give ditelluride **4** (60.8 mg, 64% yield) as a dark red oil. Found (%): C, 13.08; H, 1.20; Cl, 19.06. C_4_H_4_Cl_2_Te_2_. Calculated (%): C, 12.70; H, 1.07; Cl, 18.75. ^1^H-NMR (CDCl_3_, δ, ppm): 6.49 (d, 2H, CHCl, *J* = 13.6 Hz), 7.37 (d, 2H, TeCH, *J* = 13.6 Hz). ^13^C-NMR (CDCl_3_, δ, ppm): 93.28 (TeCH), 125.34 (CHCl). MS (70 eV), *m/z* (*I*_rel__._, %): 380 (64) [M]^+^, 319 (16) [M-C_2_H_2_Cl]^+^, 293 (27) [Te_2_Cl]^+^, 258 (18) [Te_2_]^+^, 252 (47) [M-Te]^+^, 191 (100) [TeC_2_H_2_Cl]^+^, 165 (88) [TeC_2_H]^+^, 130 (80) [Te]^+^, 61 (44) [C_2_H_2_Cl]^+^, 51 (33) [C_4_H_3_]^+^.

*E*,*E-Bis(2-chlorovinyl) telluride *(**5**). A solution of Na_2_S_2_O_5_ (0.95 g, 5 mmol) in water (5 mL) was added to a mixture of compound **1** (0.16 g, 0.5 mmol) and benzene (2 mL). The resulting mixture was vigorously stirred at room temperature for 24 h under argon. The mixture was extracted with benzene (3 × 5 mL), the organic phase was dried, filtered and the solvent was evaporated. The residue was subjected to column chromatography (eluent–hexane) to give telluride **5** (108 mg, 86% yield) as a dark yellow oil. Found (%): C, 18.94; H, 1.75; Cl, 28.68. C_4_H_4_Cl_2_Te. Calculated (%): C, 19.17; H, 1.61; Cl, 28.30. ^1^H-NMR (CDCl_3_, δ, ppm): 6.34 (d, 2H, CHCl, *J* = 14.0 Hz), 6.89 (d, 2H, TeCH, *J* = 14.0 Hz). ^13^C-NMR (CDCl_3_, δ, ppm): 100.02 (TeCH), 128.14 (CHCl).

*E*,*E-Bis(2-chlorovinyl)tellurium dibromide *(**6**). A solution of bromine (320 mg, 2 mmol) in hexane (2 mL) was added dropwise to a stirred solution of compound **5** (501 mg, 2 mmol) in hexane (5 mL) at 0 °C (an ace bath). The mixture was stirred for 1 h at 0 °C and for 1 h at room temperature. The solvents were decanted and the precipitate was washed with cold hexane and dried under vacuum to give compound **6** (755 mg, 92% yield) as a brown powder, m.p. 265–268 °C. Found (%): C, 11.46; H, 1.08; Cl, 16.97; Br, 39.23. C_4_H_4_Cl_2_Br_2_Te. Calculated (%): C, 11.71; H, 0.98; Cl, 17.28; Br, 38.94. ^1^H-NMR (DMSO-*d_6_*, δ, ppm): 7.33 (d, 2H, *J* = 13.9 Hz), 7.67 (d, 2H, *J* = 13.9 Hz). ^13^C-NMR (DMSO-*d_6_*, δ, ppm): 126.12 (TeCH), 133.70 (CHCl).

## 4. Conclusions

Convenient methods for preparation of the previously unknown compounds **1**–**6**, prospective precursors and synthons for organic synthesis, have been elaborated. The methods are efficient, simple and based on readily available starting materials (acetylene and tellurium tetrachloride). In contrast to the *syn-*addition of tellurium tetrachloride to substituted acetylenes [9,10,11,12,13,14,15] the reaction of tellurium tetrachloride with acetylene proceeds in a stereospecific manner *via* *anti*-addition to give the products **1** and **2** of *E*-stereochemistry (Scheme 2 and Scheme 3). This are the first examples of *anti*-additions of tellurium tetrachloride to acetylenic hydrocarbons. A mechanism involving the formation of the 3-membered intermediates **B** and **C** was proposed in order to explain the stereospecific *anti*-addition (Scheme 4). In the case of acetylene, the 3-membered intermediates (**B** and **C**, Scheme 4) are supposed to be energetically preferred in comparison with the 4-membered transition state **A** (Scheme 1). 

## References

[1] Schwarz K., Foltz C.M. (1957). Selenium as an integral part of factor 3 against dietary necrotic liver degeneration. J. Am. Chem. Soc..

[2] Nogueira C.W., Zeni G., Rocha J.B.T. (2004). Organoselenium and organotellurium compounds: Toxicology and pharmacology. Chem. Rev..

[3] Tiekink E.R.T. (2012). Therapeutic potential of selenium and tellurium compounds: Opportunities yet unrealized. Dalton Trans..

[4] Wieslander E., Engman L., Svensjö E., Erlansson M., Johansson U., Linden M., Andersson C.M., Brattsand R. (1998). Antioxidative properties of organotellurium compounds in cell systems. Biochem. Pharmacol..

[5] Garberg P., Engman L., Tolmachev V., Lundqvist H., Gerdes R.G., Cotgreave I.A. (1999). Binding of tellurium to hepatocellular selenoproteins during incubation with inorganic tellurite: Consequences for the activity of selenium-dependent glutathione peroxidase. Int. J. Biochem. Cell. Biol..

[6] Sredni B., Caspi R.R., Klein A., Kalechman Y., Danziger Y., Ya’akov M.B., Tamari T., Shalit F., Albeck M. (1987). A new immunomodulating compound (AS-101) with potential therapeutic application. Nature.

[7] Sredni B., Xu R.H., Albeck M., Gafter U., Gal R., Shani A., Tichler T., Shapira J., Bruderman I., Catane R. (1996). The protective role of the immunomodulator AS101 against chemotherapy-induced alopecia: Studies on human and animal models. Int. J. Cancer.

[8] Sredni-Kenigsbuch D., Shohat M., Shohat B., Ben-Amitai D., Chan C.C., David M. (2008). The novel tellurium immunomodulator AS101 inhibits interleukin-10 production and p38 MAPK expression in atopic dermatitis. J. Dermatol. Sci..

[9] Petragnani N., Campos M.M. (1962). Organic tellurium compounds. IV. Vinylic and ethynylic tellurium derivatives. Tetrahedron.

[10] Uemura S., Miyoshi H., Okano M. (1979). Regio- and stereospecific Z-iodo- and Z-bromochlorination of alkylphenylacetylenes via Z-chlorotelluration. Chem. Lett..

[11] Chieffi A., Menezes P.H., Comasseto J.V. (1997). Reduction of organotelluriun trichlorides with sodium borohydryde. Organometallics.

[12] Petragnani N., Mendes S.R., Silveira C.C. (2008). Tellurium tetrachloride: An improved method of preparation. Tetrahedron Lett..

[13] Cunha R.L.O.R., Zukerman-Schpector J., Caracelli I., Comasseto J.V. (2006). Revisiting the addition reaction of TeCl_4_ to alkynes: The crystal structure and docking studies of 1-chloro-2-trichlorotelluro-3-phenyl-propen-2-ol. J. Organometal. Chem..

[14] Chauhan A.K.S., Bharti S.N., Srivastava R.C., Butcher R.J., Duthie A. (2012). Stereospecific chlorotelluration of terminal acetylenes. J. Organomet. Chem..

[15] Zukerman-Schpector J., Haiduc I., Dabdoub M.J., Biazzotto J.C., Braga A.L., Dornelles L., Caracelli I. (2002). Dichloro-bis(2-chloro-2-phenyl-vinyl)Te(IV) and dibromo-bis(2-bromo-2-phenyl-vinyl)Te(IV): Supramolecular self-assembly through different π-aryl interactions. Z. Kristallogr..

[16] Braverman S., Cherkinsky M., Jana R., Kalendar Y., Sprecher M. (2010). Reaction of selenium and telluriun halides with propargyl alcohols. The regio- and stereoselectivity of addition to the triple bond. J. Phys. Org. Chem..

[17] Zeni G., Ludtke D.S., Panatieri R.B., Braga A.L. (2006). Vinylic tellurides: From preparation to their applicability in organic synthesis. Chem. Rev..

[18] Petragnani N., Stefani H.A. (2007). Tellurium in Organic Synthesis.

[19] Petragnani N., Stefani H.A. (2005). Advances in organic tellurium chemistry. Tetrahedron.

[20] Comasseto J.V., Stefani H.A., Chiefi A., Zukerman-Schpector J. (1991). Addition of organotellurium trihalides to acetylenes. Organometallics.

[21] Potapov V.A., Amosova S.V., Khangurov A.V., Petrov P.A. (1993). Synthesis of acetylenic tellurides by the iodomethane-induced reaction of dialkyl ditellurides with phenylacetylene. Phosphorus Sulfur Silicon Relat. Elem..

[22] Potapov V.A., Amosova S.V. (1996). New routes to unsaturated organoselenium and organotellurium compounds. Russ. J. Org. Chem..

[23] Potapov V.A., Amosova S.V., Shestakova V.Y., Zhnikin A.R., Petrov B.V. (1996). Synthesis of alkyl ethynyl tellurides and 1,2-bis(alkyltelluro) acetylenes by electrophilic-reagent-induced reaction of dialkyl ditellurides with acetylene. Rec. Trav. Chim..

[24] Potapov V.A., Amosova S.V., Petrov P.A. (1992). Aromatic substitution and dealkylation by alkanetellurolate anions. Tetrahedron Lett..

[25] Potapov V.A., Amosova S.V., Shestakova V.Y. (1998). Novel synthesis of unsaturated organoselenium and organotellurium compounds based on organic dichalcogenides and elemental chalcogens. Phosphorus Sulfur Silicon Relat. Elem..

[26] Potapov V.A., Amosova S.V., Beletskaya I.P., Starkova A.A., Hevesi L. (1998). Organic diselenides and ditellurides: Disproportionations, synthesis of stannyl selenides, reactions with acetylenes. Phosphorus Sulfur Silicon Relat. Elem..

[27] Potapov V.A., Trofimov B.A. (2005). 1-(Organosulfanyl)-, 1-(organoselanyl)-, and 1-(organotellanyl)alk-1-yne. Sci. Synth..

[28] Potapov V.A., Amosova S.V. (2003). New methods for preparation of organoselenium and organotellurium compounds from elemental chalcogens. Russ. J. Org. Chem..

[29] Potapov V.A., Musalov M.V., Amosova S.V., Musalova M.V., Penzik M.V. (2011). Reaction of selenium dichloride with divinyl telluride. Russ. J. Org. Chem..

[30] Musalov M.V., Potapov V.A., Amosova S.V., Musalova M.V., Volkova K.A. (2011). Reactions of selenium dichloride and dibromide with diallyl telluride. Russ. J. Gen. Chem..

[31] Trofimov B.A., Gusarova N.K., Tatarinova A.A., Potapov V.A., Sinegovskaya L.M., Amosova S.V., Voronkov M.G. (1988). Alkyl vinyl tellurides from tellurium, acetylene and alkyl halides. Tetrahedron Lett..

[32] Potapov V.A., Amosova S.V. (1993). Synthesis of vinylic selenides and tellurides by the addition of alkaneselenolate and alkanetellurolate anions to acetylenes. Phosphorus Sulfur Silicon Relat. Elem..

[33] Gusarova N.K., Trofimov B.A., Tatarinova A.A., Potapov V.A., Gusarov A.V., Amosova S.V., Voronkov M.G. (1989). Reactions of chalcogenes with acetylene .4. Synthesis of divinyl telluride by the direct reaction of tellurium with acetylene. Zh. Org. Khim..

[34] Gusarova N.K., Trofimov B.A., Tatarinova A.A., Potapov V.A., Sinegovskaya L.M., Amosova S.V., Voronkov M.G. (1988). Reactions of chalcogens with acetylene. 3. Alkyl vinyl tellurides from tellurium, acetylene and alkyl halides. Zh. Org. Khim..

[35] Trofimov B.A., Gusarova N.K., Tatarinova A.A., Amosova S.V., Sinegovskaya L.M., Keiko V.V., Potapov V.A. (1984). Vinyl methyl telluride. Zh. Org. Khim..

[36] Potapov V.A., Gusarova N.K., Amosova S.V., Tatarinova A.A., Sinegovskaya L.M., Trofimov B.A. (1986). Elementary tellurium reaction with phenylacetylene-Synthesis of 3-benzylidene-4-phenyl-1,2-ditellurole and Z,Z-distyryltelluride. Zh. Org. Khim..

[37] Potapov V.A., Amosova S.V., Kashik A.S. (1989). Reactions of selenium and tellurium metals with phenylacetylene in 3-phase catalytic systems. Tetrahedron Lett..

[38] Potapov V.A., Kashik A.S., Amosova S.V. (1988). Reaction of metal tellurium with phenylacetylene under the phase-transfer catalysis. Zh. Org. Khim..

[39] Amosova S.V., Martynov A.V., Shagun V.A., Musalov M.V., Larina L.I., Krivdin L.B., Zhilitskaya L.V., Voronkov M.G. (2008). *Anti*-markovnikov addition of tellurium tetrachloride to trimethyl ethynyl silane. J. Organomet. Chem..

[40] Amosova S.V., Martynov A.V., Penzik M.V., Makhaeva N.A., Potapov V.A., Albanov A.I., Zhilitskaya L.V., Voronkov M.G. (2008). 4,4-Diorganyl-1,1,3,6-tetrachloro-1,4-tellura(IV)silafulvenes—New class of tellurium-silicon containing heterocycles. J. Organomet. Chem..

[41] Trofimov B.A., Gusariva N.K. (2007). Acetylene: New prospects of classical reactions. Russ. Chem. Rev..

[42] Potapov V.A., Musalov M.V., Musalova M.V., Amosova S.V. (2009). Reaction of tellurium tetrachloride with acetylene. Russ. Chem. Bull..

[43] Shmid G.H., Patai S. (1978). Electrophilic Additions to Carbon-Carbon Triple Bonds. The Chemistry of the Carbon-Carbon Triple Bond.

[44] Dabdoub M.J., Dabdoub V.B., Comasseto J.V., Petragnani N. (1986). Synthesis of vinylic tellurides. J. Organomet. Chem..

[45] Dabdoub M.J., Comasseto J.V. (1988). Divinyl ditelluride: Synthesis and reactivity. J. Organomet. Chem..

[46] Amosova S.V., Gostevskaya V.I., Gavrilova G.M., Potapov V.A., Kashik A.S. (1988). Preparative synthesis of divinyl ditelluride. Zh. Org. Khim..

